# Bacterial and protozoal agents of canine vector-borne diseases in the blood of domestic and stray dogs from southern Portugal

**DOI:** 10.1186/s13071-015-0759-8

**Published:** 2015-03-23

**Authors:** Carla Maia, Bruno Almeida, Mónica Coimbra, Maria Catarina Fernandes, José Manuel Cristóvão, Cláudia Ramos, Ângela Martins, Filipe Martinho, Pedro Silva, Nuno Neves, Mónica Nunes, Maria Luísa Vieira, Luís Cardoso, Lenea Campino

**Affiliations:** Unidade de Parasitologia Médica, Instituto de Higiene e Medicina Tropical (IHMT), Universidade Nova de Lisboa (UNL), Lisbon, Portugal; Global Health and Tropical Medicine, IHMT-UNL, Lisbon, Portugal; Faculdade de Medicina Veterinária, Universidade Lusófona de Humanidades e Tecnologias, Lisbon, Portugal; Clínica Veterinária Porto Seguro, Olhão, Portugal; Hospital Veterinário da Arrábida, Azeitão, Portugal; Nyctea Lda., Lisbon, Portugal; Vetévora, Évora, Portugal; Clube Animal, Beja, Portugal; Unidade de Microbiologia Médica, IHMT-UNL, Lisbon, Portugal; Department of Veterinary Sciences, School of Agrarian and Veterinary Sciences, University of Trás-os-Montes e Alto Douro, Vila Real, Portugal; Departamento de Ciências Biomédicas e Medicina, Universidade do Algarve, Faro, Portugal

**Keywords:** Dogs, Canine vector-borne diseases, Bacteria, Protozoa, Portugal, Polymerase chain reaction

## Abstract

**Background:**

The so-called canine vector-borne diseases (CVBD) are caused by a wide range of pathogens transmitted by arthropods. In addition to their veterinary importance, many of these canine vector-borne pathogens can also affect the human population due to their zoonotic potential, a situation that requires a One Health approach. As the prevalence of vector-borne pathogens in cats from southern Portugal has been recently evaluated, the aim of the present study was to assess if the same agents were present in dogs living in the same area, and to assess positivity-associated risk factors.

**Methods:**

One thousand and ten dogs (521 domestic and 489 stray) from veterinary medical centres and animal shelters in southern Portugal were enrolled. *Anaplasma* spp./*Ehrlichia* spp., *Bartonella* spp., *Borrelia burgdorferi* sensu lato, *Babesia* spp., *Hepatozoon* spp. and *Leishmania infantum* infections were evaluated by polymerase chain reaction (PCR) assays in blood samples.

**Results:**

Sixty-eight (6.7%) dogs were PCR-positive to at least one of the tested CVBD agent species, genera or complex, including one dog found positive to two different genera. Nineteen (1.9%) dogs were positive to *Anaplasma* spp./*Ehrlichia* spp., eight (0.8%) to *B. burgdorferi* s.l., 31 (3.1%) to *Hepatozoon* spp. and 11 (1.1%) to *L. infantum. Anaplasma platys*, *Ehrlichia canis*, *B. burgdorferi*s.l. and *Hepatozoon canis* were identified by DNA sequencing, including one animal confirmed with both *A. platys* and *H. canis*. Furthermore, *Wolbachia* spp. was amplified in blood from four dogs. None of the tested dogs was positive by PCR for *Bartonella* spp. or *Babesia* spp.

**Conclusions:**

The molecular identification of CVBD agents in southern Portugal, some of them with zoonotic concern, reinforces the importance to alert the veterinary community, owners and public health authorities to prevent the risk of transmission of vector-borne pathogens among dogs and to other vertebrate hosts including humans. The prevalence of the selected pathogens was lower than that previously found in cats from the same region, probably because veterinarians and owners are more aware of them in the canine population and control measures are used more often.

## Background

Canine vector-borne diseases (CVBD) comprise a group of globally distributed and spreading illnesses that are caused by a wide range of pathogens transmitted by arthropods [1-4]. In addition to their veterinary importance, many of these canine vector-borne pathogens can also affect the human population due to their zoonotic potential, a situation that requires a One Health approach [5,6].

*Anaplasma phagocytophilum* and *Anaplasma platys* cause canine granulocytic anaplasmosis and infectious canine cyclic thrombocytopenia, respectively. Both agents can infect a range of domestic and wild vertebrate hosts, including dogs and humans [7-10]. *A. phagocytophilum* is transmitted by ticks of the genus *Ixodes* and *A. platys* presumably by the *Rhipicephalus sanguineus* ticks. In Portugal *A. platys* DNA has been detected in clinically suspect dogs living in the north and south of Portugal [11,12], while the overall national seroprevalence of *Anaplasma* spp. has ranged from 4.5% in apparently healthy to 9.2% in clinically suspect dogs [3]. *Ehrlichia canis* (transmitted by *R. sanguineus*) is a causative agent of acute or chronic canine monocytic ehrlichiosis. *E. canis* has been molecularly detected in dogs from the north [12,13] and from the south of Portugal [14]. Seroprevalence at the national level ranged from 4.1% in apparently healthy dogs to 16.4% in animals clinically suspected of a CVBD [3].

Seven *Bartonella* species transmitted by several arthropod vectors, including fleas and *Ixodes* spp. ticks, have been implicated as canine pathogens [15]. To date, no dog with *Bartonella* spp. infection has been reported in Portugal. Spirochetes belonging to the *Borrelia burgdorferi* sensu lato complex are the agents of Lyme borreliosis. In Europe, *B. burgdorferi* s.l. is mainly transmitted by *I. ricinus* [16]. Though few infected dogs show similar clinical signs, most of them are subclinical hosts [17] and can be sentinels for this infection. In Portugal, seropositivity to *B. burgdorferi* s.l. has ranged from 0.2% in apparently healthy dogs to 0.5% in clinical suspected animals in a countrywide investigation [3].

Canine piroplasmosis or babesiosis, mainly caused by several *Babesia* spp. haemoparasites, is a protozoal tick-borne disease with worldwide distribution [18]. *Babesia canis* (transmitted by *Dermacentor reticulatus*), *Babesia vogeli* (transmitted by *R. sanguineus*) and the *Babesia microti*-like piroplasm (syn. *Theileria annae*) were molecularly confirmed for the first time in Portugal in dogs from the north of the country [19,20]. Canine hepatozoonosis caused by the protozoan *Hepatozoon canis* transmitted by the ingestion of *R. sanguineus* is a common infection of dogs from the Old World [21]. *H. canis* has already been molecularly detected in dogs from the north [13,22] and from the south of Portugal [23]. Canine leishmaniosis (CanL), a zoonotic disease endemic in southern Europe is caused by the protozoan *L. infantum* transmitted by *Phlebotomus* spp. sand flies [24]. CanL is endemic in Portugal, with an overall national seroprevalence of 6.3% [25].

As the prevalence of vector-borne pathogens in cats from southern Portugal was recently evaluated [26], the aim of the present study was to assess if the same agents with veterinary and zoonotic importance were present in dogs living in the same region, and to assess positivity-associated risk factors.

## Methods

### Animals and samples

From December 2011 to April 2014, a total of 1,010 dogs (521 domestic and 489 stray), from veterinary medical centres and animal shelters in southern Portugal, were studied (Table 1). Animals were from the districts of Lisbon (n = 305), Setúbal (n = 453, which include 24 dogs from the contiguous districts of Évora and Beja) and Faro (n = 252).

**Table 1:** **Prevalence of vector-borne pathogen species, gender or complex as detected by PCR in 1,010 dogsfrom southern Portugal**

**Variable//category**	**N° of characterized dogs (%)**	**N° of positive dogs (%)**				
		***Anaplasma*** **/** ***Ehrlichia***	***B. burgdorferi*** **s.l.**	***Hepatozoon***	***L. infantum***	**≥1 pathogen**
**Region**	1,010					
Lisboa	305 (30.2)	1 (0.3)^a^	1 (0.3)	2 (0.7)^a^	7 (2.3)	11 (3.6)^a^
Setúbal	453 (44.9)	2 (0.4)^b^	3 (0.7)	10 (2.2)^b^	3 (0.7)	17 (3.8)^b^
Algarve	252 (25.0)	16 (6.3)^a,b^	4 (1.6)	19 (7.5)^a,b^	1 (0.4)	40 (15.9)^a,b^
**Breed**	793					
Defined	344 (43.4)	4 (1.2)	3 (0.9)	8 (2.3)	6 (1.7)	21 (6.1)
Mongrel	449 (56.6)	12 (2.7)	5 (1.1)	18 (4.0)	4 (0.9)	39 (8.7)
**Gender**	1,004					
Female	504 (50.2)	9 (1.8)	5 (1.0)	15 (3.0)	4 (0.8)	33 (6.5)
Male	500 (49.8)	9 (1.8)	3 (0.6)	14 (2.8)	7 (1.4)	33 (6.6)
**Age** (months)	938					
[1-11]	73 (7.8)	3 (4.1)	2 (2.7)	0 (0.0)	0 (0.0)	5 (6.8)
[12–83]	576 (61.4)	10 (1.7)	4 (0.7)	15 (2.6)	7 (1.2)	36 (6.3)
[84–228]	289 (30.8)	3 (1.0)	1 (0.3)	7 (2.4)	3 (1.0)	14 (4.8)
**Lifestyle**	1,010					
Domestic	521 (51.6)	15 (2.9)^a^	6 (1.2)	19 (3.6)	6 (1.2)	45 (8.6)^a^
Stray	489 (48.4)	4 (0.8)^a^	2 (0.4)	12 (2.5)	5 (1.0)	23 (4.7)^a^
**Housing**	852					
Indoors	63 (7.4)	0 (0.0)	0 (0.0)	0 (0.0)	0 (0.0)	0 (0.0)^a,b^
Mixed	182 (21.4)	5 (2.7)	3 (1.6)	11 (6.0)	0 (0.0)	19 (10.4)^a^
Outdoors	607 (71.2)	11 (1.8)	4 (0.7)	18 (3.0)	5 (0.8)	38 (6.3)^b^
**Acaricides-insecticides**	963					
Yes	448 (46.5)	5 (1.5)	4 (0.8)	10 (2.2)	6 (1.3)	25 (5.6)
No	515 (53.5)	12 (2.3)	4 (0.8)	19 (3.7)	4 (0.8)	38 (7.4)
**Clinical status**	906					
Non-suspect	700 (77.3)	12 (1.7)	5 (0.7)	26 (3.7)	7 (1.0)	49 (7.0)
Suspect	206 (22.7)	6 (2.9)	2 (1.0)	3 (1.5)	3 (1.5)	14 (6.8)
**Total**	1,010	19 (1.9)	8 (0.8)	31 (3.1)	11 (1.1)	68 (6.7)

^a,b^Statistically significant difference for the same agent between categories of the same variable (*p* < 0.05).

Domestic dogs were randomly included after owners’ informed consent. Consent for enrolment of stray dogs was obtained from the person in charge of each shelter. Out of the 489 stray animals, 457 were sheltered for adoption, and 32 others were captured and euthanized in the scope of official animal control programs.

Whole blood samples (1–2 ml) were collected by cephalic or jugular venipuncture and spotted onto filter paper for DNA extraction. Samples were dried at room temperature and kept at 4°C until tested. Whenever available, data on the region, breed, gender, age, living conditions, use of acaricides/insecticides and clinical status (presence or absence of signs compatible with a CVBD) were registered for each dog (Table 1). Clinical signs comprised anorexia, muscular atrophy, dermatological manifestations, epistaxis, exercise intolerance, fever, gastrointestinal alterations, lameness, lethargy, lymphadenopathy, onychogryphosis, ocular manifestations, pale mucous or weight loss.

This study was ethically approved by the boards of the IHMT-UNL and of the Faculty of Veterinary Medicine (ULHT) as complying with the Portuguese legislation for the protection of animals (Law no. 92/1995).

### PCR amplification and DNA sequencing

A commercial kit (Kit Citogene®, Citomed, Portugal) was used to extract DNA from blood on filter paper. Four discs of filter paper (4 mm in diameter each) were incubated with lysis buffer (150 μl) and 1.5 μl of proteinase K (20 mg/ml). Further DNA extraction followed the kit manufacturer’s instructions.

Positivity to *Anaplasma* spp./*Ehrlichia* spp., *Bartonella* spp., *B. burgdorferi* s.l., *Babesia* spp., *Hepatozoon* spp. and *L. infantum* DNA in blood samples was tested by PCR according to previously described protocols (Table 2). PCR amplifications were performed in a 25 μl final volume containing 12.5 μl of NZYTaq 2x Green Master Mix (Nyztech, Portugal), 1 μl of each primer (10 pmol) and 3 μl of DNA template. In all amplifications a positive control containing genomic target DNA and a negative control without DNA were included. The reaction mixtures were cycled in a Thermo Electron Corporation® Px2 Termal Cycler (VWR, USA). PCR products were visualized under UV illumination after electrophoresis migration on a 1.5% gel agarose stained with GreenSafe Premium® (Nzytech), using a 100 bp DNA ladder as a marker.

**Table 2:** **Primers sets for PCR amplification of CVBD agents**

**Pathogen**	**Primers**	**Product size (bp)**	**Reference**
*Anaplasma* spp./*Ehrlichia* spp.	EHR16SD: 5'-GGT ACC YAC AGA AGA AGTCC-3'	345	[27]
	EHR16SR: 5'-TAG CAC TCA TCG TTT ACAGC-3'		
*Bartonella* spp.	325 s: 5'-CTTCAGATGATGATCCCAAGCCTTCTGGCG-3'	500-800	[28]
	1100as: 5'-GAACCGACGACCCCCTGCTTGCAAAGCA-3'		
*Borrelia burgdorferi* s.l.	Outer primers:	774	[29]
	132f: 5'-TGGTATGGGAGTTTCTGG-3'		
	905r: 5'-TCTGTCATTGTAGCATCTTT-3'		
	Inner primers:	604	[29]
	220f: 5'-CAGACAACAGAGGGAAAT-3'		
	823r: 5'-TCAAGTCTATTTTGGAAAGCACC-3'		
*Babesia* spp.	PIRO-A: 5'-AAT ACC CAA TCC TGA CACAGG G-3'	400	[27]
	PIRO-B: 5'-TTA AAT ACG AAT GCC CCCAAC-3'		
*Hepatozoon* spp.	HEP-F: 5'-ATA CAT GAG CAA AAT CTC AAC-3'	626-666	[30]
	HEP-R: 5'-CTT ATT ATT CCA TGC TGC AG-3'		
*Leishmania infantum*	MC1: 5’-GTTAGCCGATGGTGGTCTTG-3’	447	[31]
	MC2: 5’-CACCCATTTTTCCGATTTTG-3’		

PCR products were purified with a High Pure PCR Product Purification Kit (Roche®, Germany) according to the manufacturer’s instructions and directly sequenced (one direction) (Stabvida®, Portugal), using the same primers as those used for the DNA amplification. Species identity of the obtained sequences was determined according to the closest BLAST match (identity ≥ 99% for the first 30 matches) to a GenBank® accession and deposited in DNA Data Bank of Japan (DDBJ) (http://www.DDBJ.nig.ac.jp).

### Statistical analysis

Percentages of positivity to CVBD agents were compared by the Chi-square or Fisher’s exact tests. A *p* value < 0.05 was considered as statistically significant. The exact binomial test was used to calculate confidence intervals for the proportions, with a 95% confidence level (CI). Analyses were performed with SPSS® 21 software for Windows and with StatLib.

## Results

Sixty-eight (6.7%; CI: 5.3-8.5%) dogs were PCR-positive to at least one of the tested species, genera or complex of CVBD agents (Table 1). Nineteen (1.9%; CI: 1.1-2.9%) dogs were positive to *Anaplasma* spp./*Ehrlichia* spp., eight (0.8%; CI: 0.3-1.5%) to *B. burgdorferi* s.l., 31 (3.1%; CI: 2.1-4.3) to *Hepatozoon* spp. and 11(1.1%; CI: 0.5-1.9) to *L. infantum* (Table 3). *Wolbachia* spp. DNA (amplified with the same primers used to detect *Anaplasma* spp./*Ehrlichia* spp.) was detected in four dogs, while DNA of *Bartonella* spp. or *Babesia* spp. was not amplified from any dog in the study.

**Table 3:** **Single and mixed PCR-positivity to species, genera and/or complex of CVBD agents in 1,010 dogs from southern Portugal**

**Agents**	**No. positive dogs (%)**	**DDBJ accessions**
**Single infections**	67 (6.6)	
*Anaplasma* spp./*Ehrlichia* spp.	18 (1.8)	
[*Anaplasma platys*]	[4 (0.4)]	[LC018179 to LC018182]
[*Ehrlichia canis*]	[5 (0.5)]	[LC018184 to LC018188]
*Borrelia burgdorferi* s.l.	8 (0.8)	[LC018211 to LC018216]
*Hepatozoon* spp.	30 (3.0)	
[*Hepatozoon canis*]	[17 (1.7)]	[LC018193 to LC018209]
*Leishmania infantum*	11 (1.1)	
**Co-infections**	1 (0.1)	
*Anaplasma* spp./*Ehrlichia* spp. and *Hepatozoon* spp.	1 (0.1)	
[*A. platys* and *H. canis*]	[1 (0.1)]	[LC018183 and LC018210]
**Total**	68 (6.7)	

As shown in Table 1, the prevalence of *Anaplasma* spp/*Ehrlichia* spp. was statistically higher in domestic dogs. Positivity to these bacteria and to *Hepatozoon* spp. was higher in dogs living in the Algarve region. Statistically significant differences were also found for PCR positivity to at least one of the studied agents in domestic dogs, in dogs with access to outdoors and in dogs living in the Algarve region.

Sequencing confirmed *A. platys* in five, *E. canis* in five, *B. burgdorferi*s.l. in six and *H. canis* in 18 dogs, including one animal with both *A. platys* and *H. canis* (Table 3); and revealed *Wolbachia* spp. (DDBJ accessions: LC018189 to LC018192) in four dogs.

## Discussion

This is the most comprehensive study carried out in dogs from southern Portugal on the prevalence of infection with CVBD agents as it included domestic and stray animals with and without clinical signs compatible with a vector-borne disease. DNA from these pathogens taken all together was less frequently detected in dogs (6.7%; *p* < 0.001) than in cats (29.9%; 194/649) from the same region [26]. Furthermore, only one (0.1%) dog was found co-infected (with two pathogens), whereas 29 (4.5%) cats were positive to two agents and four (0.6%) cats to three agents [26].

In this study *A. platys* has been molecularly confirmed to infect dogs from the south of the country, corroborating previous detection of this bacterium in dogs [11,23] and in *R. sanguineus* [32] from the same region. The prevalence of positivity to *Anaplasma*/*Ehrlichia* in this work (1.9%) was lower than the 4.0% obtained in Spain [33] and the 3.7-6.0% in Italy [34], which might be related with the targeted population. In fact, in the works of Tabar et al. [33] and Trotta et al. [34], all the positive dogs were sick animals with clinical signs compatible with vector-borne diseases and admitted for medical treatment, while in the present work most of the enrolled animals were apparently healthy. Interestingly, in our study most of the animals harbouring *Anaplasma*/*Ehrlichia* DNA were from Faro, overlapping the Algarve region, the southern most district of continental Portugal, which seem to follow the trend revealed by Cardoso et al. [3] that the prevalence of antibodies against *Anaplasma* spp. and *E. canis* in dogs from southern Portugal was significantly higher than in dogs from the northern and central regions of the country.

In the present work, *B. burgdorferi* s.l. DNA was amplified from 0.8% of the screened animals, providing the first molecular evidence of naturally occurring *B. burgdorferi* s.l. infection in dogs from Portugal. The exposure of dogs to these spirochetes was previously demonstrated by specific serology in the Algarve [35] and in the Alentejo and Lisbon regions [3]. Furthermore, *B. burgdorferi* s.l. genospecies, *Borrelia lusitaniae* was isolated from humans [36-38] and DNA of *B. burgdorferi* s.l. was detected in ticks [32,39] and cats from the south of the country [26]. Nevertheless, information on the clinical signs associated with *Borrelia* infections in dogs and their role as sentinels is still limited [6].

*H. canis* was the most prevalent pathogen detected in all the assessed dogs, with a significantly higher prevalence in animals living in the Algarve. In fact, *H. canis* has recently been identified in dogs [23], in *R. sanguineus* collected from dogs living in this region [32], and also in foxes from the south [40], showing that the protozoan is widespread in this area of the country. Although in this study only three out of the 31 infected dogs presented clinical signs, subclinical infections should not be neglected as they may progress to a severe disease and warrant treatment [41]. Concurrent infections of *H. canis* with other canine pathogens are common [21]; however, in the present work only one animal apparently healthy was co-infected with *A. platys* and the protozoan. Although this individual dog had no clinical signs of a CVBD, co-infections may potentiate disease pathogenesis, altering clinical manifestations associated with single infections [42].

The overall prevalence of *L. infantum* infection in the present study (1.1%) was much lower (*p* < 0.001) than the 34.9% obtained in 152 dogs from Lisbon [43]. The lower detection of *Leishmania* DNA might be due to the: (i) dynamics of infection over time, which may depend on the abundance and distribution of the proven vector species in conjunction with the number of infected vertebrate hosts [44], and (ii) insufficient data regarding the duration of parasitaemia in infected dogs. In fact, and taking into account a seroprevalence of 18.2% recently obtained in 170 dogs from the Algarve region [45], PCR with blood should be used to complement serological results and not only by itself to detect *Leishmania* infection, as it can lead to false negative results, especially in subclinically infected dogs [46].

PCR-positivity to one or more genera/complex of CVBD agents was found to be associated with domestic dogs, with animals living in the Algarve and with an outdoor or mixed (i.e. with outdoor access) housing. In fact, most of the domestic dogs harbouring DNA of the studied pathogens lived in rural areas from the Algarve region and used to spend most of their time exclusively outdoors, thus increasing their exposure to arthropod vectors and the agents they might transmit.

The role of domestic dogs as reservoirs of *Bartonella* spp. is less clear than for cats, and the former are probably accidental hosts. Nevertheless, they are excellent sentinels for human infections because a similar disease spectrum develops in dogs [47]. Serologic and molecular evidence of *Bartonella henselae* and *Bartonella clarridgeiae* exist for cats from the south of Portugal [26,48]. Thus, the non-detection of *Bartonella* DNA in the present study might be related with differences in immune responses, host preference of particular vectors or innate resistance in dogs to these bacteria. On the other hand, the definitive diagnosis of *Bartonella* infection is challenging due to the fastidious nature and intracellular tropism of these bacteria for erythrocytes and endothelial cells [49]. According to Perez et al. [50], enrichment culture and subculture, followed by PCR amplification, enhances molecular diagnostic sensitivity in dogs. Thus, it is possible that the PCR done directly from blood samples might have missed some positive cases; nevertheless, the prevalence of infection at the population level, if any, must be very low.

Albeit the detection of *B. canis*, *B. vogeli* and the *B. microti*-like piroplasm has already been reported in dogs from the north of Portugal [13,19,20,22] and *B. vogeli* in dogs from the south of the country [23], in the present study none of the screened animals harboured piroplasmid DNA. The non-detection of *B. canis* could somehow be expected as its vector, *D. reticulatus*, is more abundant in the north of the country. However, the absence of *B. vogeli* and *B. microti*-like DNA is more difficult to explain, since both have already been detected in southern Portugal, the former in cats [26], dogs [23] and ticks [32], and the latter in foxes [51]. According to a recent questionnaire-based survey on the distribution of canine babesiosis in western Europe, the annual incidence of this parasitosis in southern Spain, which is geographically close to the area surveyed in this study, was estimated to be 0.0-0.7% [52]. Furthermore, a 58% prevalence of antibodies anti-*Babesia* spp. was reported among 331 dogs from kennels/shelters in southern Portugal [53]. The absence of *Babesia* spp. infection in the present study might be related with differences in the genetic background/immune system or between vector-dog interactions. Further studies are needed to better understand the epidemiological importance of these findings.

## Conclusion

The identification of CVBD agents in southern Portugal, some of them with zoonotic concern, reinforces the importance to alert the veterinary community, owners and public health authorities to prevent the risk of transmission of vector-borne pathogens among dogs and to other vertebrate hosts including humans. Interestingly, the prevalence of the selected pathogens was much lower than that previously found in cats from the same region [26], probably because veterinarians and owners are much aware of them in the canine population and prophylatic measures such as insecticides and acaricides are used.
